# Genome-wide identification, evolution and expression of the *CPP* gene family in six Theaceae species

**DOI:** 10.3389/fpls.2025.1700390

**Published:** 2025-11-10

**Authors:** Wei Zheng, Chunling Yang, Wenyang Li, Wanmeng Bu, Fujun Bu

**Affiliations:** 1 College of Forestry, Xinyang Agriculture and Forestry University, Xinyang, China; 2 Henan Dabieshan National Field Observation and Research Station of Forest Ecosystem, Zhengzhou, China; 3 Xinyang Academy of Ecological Research, Xinyang, China; 4 College of Agriculture, Xinyang Agriculture and Forestry University, Xinyang, China; 5 College of Foreign Languages, Xinyang Agriculture and Forestry University, Xinyang, China; 6 Xinyang Forestry Science Research Institute, Xinyang, China

**Keywords:** CPP gene family, Theaceae, evolution, abiotic stress, *Camellia oleifera*

## Abstract

The Cysteine-rich polycomb-like protein (CPP) gene family encodes transcription factors that function as key regulators in various plant processes, including growth, development, and responses to environmental stresses. However, systematic analysis of this gene family in Theaceae plants remains limited. In this study, we comprehensively identified and analyzed the *CPP* gene family in six Theaceae species, revealing a total of 65 members that were phylogenetically classified into two distinct subfamilies. Multiple sequence alignment revealed that all CPP proteins contain conserved CXC domains (C1 and C2) and an intervening R motif. Gene structure analysis indicated that Class II genes are more conserved, with a predominant structure of 8 exons (71% of members). In contrast, Class I genes most contained 10 exons (48.4%). Codon usage bias analysis identified two distinct groups: 22 codons with high usage frequency and 42 with low usage. Collinearity analysis suggested that whole-genome duplication was the primary driver of the expansion of the *CPP* gene family, with no tandem duplications detected. A total of 82 types of cis-regulatory elements were identified, with stress-responsive elements being the most abundant. Transcriptome analysis showed that Class I *CPP* genes, such as *CsinCPP2*, *CcheCPP1*, and *ColeCPP12*, had high expression in leaves, apical buds, and stems. Several Class II *CPP* genes, such as *ColeCPP1*, *CsinCPP9*, *ColeCPP2*, and *CcheCPP8*, were significantly upregulated in multiple stress. qRT-PCR expression profiling under drought and salt stress in *Camellia oleifera* yielded results consistent with the transcriptome data. This study provides a comprehensive and detailed analysis of the *CPP* gene family in Theaceae, offering valuable insights into the evolutionary dynamics and functional diversification of these genes.

## 1 Introduction

The *CPP* (cysteine-rich polycomb-like protein) transcription factors, also referred to as *TCX* (tesmin/TSO1-like CXC protein) transcription factors, are widely distributed in animals and plants. This relatively small transcription factor family (Yang et al., 2008) is characterized by one or two cysteine-rich CXC domains (Wang, 2010), which mediate DNA binding and target gene regulation (Lu et al., 2013). Based on the characteristics of the *CXC* domain, genome-wide identification and analysis of the *CPP* gene family have been conducted in several plants, including *Arabidopsis thaliana* (Yang et al., 2008), *Oryza sativa* (Almeida et al., 2017; Yang et al., 2008), *Zea mays* (Song et al., 2016), *Cucumis sativus* (Zhou et al., 2018), *Camellia sinensis* (Yang et al., 2019), *Triticum aestivum* L (Ullah et al., 2022), *Solanum lycopersicum* (Sun et al., 2023) and *Malus domestica* (Jiang et al., 2025). Subcellular-localization predictions indicated that all CPP proteins are predominantly localized to the nucleus, aligning with their canonical role as transcription factors (Nan et al., 2021).

The first identified *CPP* gene was *TSO1* in *A. thaliana*, which primarily regulates cell division and plays a key role in flowering (Hauser et al., 2000; Sijacic et al., 2011). Additionally, *TSO1* has been found to modulate root and shoot development during seed germination by interacting with *MYB* protein (Wang et al., 2018). *CPP* transcription factors exhibit specific expression in the symbiotic nodule tissues of *Glycine max*, where they participate in nodule growth regulation (Hauser et al., 2000). Studies have demonstrated that *CPP* transcription factors are involved in various hormone signaling pathways and stress responses (Zhou et al., 2018) (Ullah et al., 2022). For instance, abscisic acid suppresses the expression of cucumber genes *CsCPP01*, *CsCPP02*, *CsCPP04*, and *CsCPP05*, aiding plants in adapting to environmental stresses and enhancing stress tolerance (Zhou et al., 2018). In *Medicago truncatula*, the expression of *MtCPP2* and *MtCPP8* genes increases under salt stress, highlighting their role in the salt stress response (Tian et al., 2022). In *Zea mays*, *ZmCPP* genes show differential expression in response to cold, heat, drought, and salt stress, indicating their participation in diverse stress response processes (Song et al., 2016).

In this study, we comprehensively identify and characterize the *CPP* gene family in six Theaceae species, including phylogenetic relationships, gene structure, protein structure, codon bias, chromosomal distribution, homology relationships, and promoter and cis-element analysis. We further explored the expression patterns of *CPP* genes across multiple tissues and conditions, with particular focus on validating the expression patterns of *C. oleifera* under drought and salt stress conditions. This comprehensive analysis will provide insights into the structural and functional conservation of the *CPP* gene family in Theaceae plants and contribute to understanding their adaptive evolution in response to abiotic stress.

## 2 Materials and methods

### 2.1 Experimental materials and treatments

Experimental materials used *C. oleifera* var. ‘Changlin No. 4’ variety, selecting two-year-old seedlings with uniform growth seedling. The cultivation substrate consisted of forest topsoil, river sand, and decomposed sawdust mixed at a ratio of 3:1:1. Twenty-four pots, each 15 cm in diameter and 20 cm in height, were used, with a 12-hour photoperiod. Drought stress was simulated by controlled irrigation using a soil moisture rapid tester (Model: ST-WSY, Shandong Santi Hongke Co., Ltd., China). Plants under normal watering, maintained at 70-80% soil relative water content, served as the control group (CK). Leaf tissues were collected at 24, 48, and 72 h after irrigation cessation for the drought treatment. For the salt stress treatment, 500 mL of 200 mg/L NaCl solution (Liu et al., 2021) was applied per pot, while control groups received 500 mL of sterile water. Leaf samples were collected at 24, 48, and 72 h after salt treatment. Both drought and salt stress treatments included three biological replicates. All collected leaf materials were immediately frozen with liquid nitrogen and stored at -80°C.

### 2.2 *CPP* gene family member identification and physicochemical property analysis

Genomic and proteomic data for six Theaceae species—*Camellia chekiangoleosa*, *Camellia oleifera*, *Camellia sinensis*, *Camellia crapnelliana*, *Camellia japonica*, and *Stewartia sinensis*—were retrieved from the Tea Plant Information Archive (TPIA, https://tpia.teaplants.cn/index.html). The *CPP* family HMM model (PF03638) was downloaded from the Pfam database (https://www.ebi.ac.uk/interpro/entry/pfam/PF03638/). Using HMMER (v3.3.2) (Potter et al., 2018) (with the incdomE parameter set to 0.01), we searched for *CPP* conserved domains in protein sequences across all species. Additionally, we verified the presence of *CPP* domains using InterProScan (https://www.ebi.ac.uk/interpro/search/sequence/) and the CDD database (Marchler-Bauer et al., 2015) (https://www.ncbi.nlm.nih.gov/Structure/bwrpsb/bwrpsb.cgi). The Peptides package (v2.4.6) pI/Mw tool was used to estimate physicochemical parameters (molecular weight [MW] and isoelectric point [pI]). In-silico subcellular localization was predicted using WoLF PSORT (https://wolfpsort.hgc.jp/). Chromosomal locations, gene length information, and exon details were recorded in **Supplementary Table S1**.

### 2.3 CPP protein family phylogenetic analysis

MUSCLE software (v5.1) (Edgar, 2004) was used for multiple sequence alignment of full-length CPP protein sequences from six Theaceae species plus with previous identified *Oryza sativa* and *Arabidopsis thalian CPP* genes (Lu et al., 2013) with default parameters. After alignment, a phylogenetic tree was constructed with RAxML (v8.2.12) (Stamatakis, 2014) under the Maximum Likelihood (ML) criterion. The JTT+G model, selected by ModelFinder (Kalyaanamoorthy et al., 2017), was used with 1000 bootstrap replicates. The resulting phylogenetic tree was visualized and edited using the iTOL platform (https://itol.embl.de/).

### 2.4 Gene structure and motif analysis of the *CPP* gene family

Exon-intron structure information for the *CPP* gene family was obtained from GFF files and genome data. Using the motif prediction tool MEME (v.5.5.7) (Bailey et al., 2009), we investigated conserved motifs in the *CPP* protein family. The maximum number of motifs was set to 10, while other parameters remained at default values.

### 2.5 Codon preference analysis of the *CPP* gene family

Using EMBOSS (v.6.6.0.0, http://www.bioinformatics.nl/emboss-explorer/) and CODONW (v.1.4.4, https://sourceforge.net/projects/codonw/files/codonw/), we calculated Codon Adaptation Index (CAI), Effective Number of Codons (ENC), and total Guanine-Cytosine (GC) content, along with GC content at the first position (GC1), GC content at the second position (GC2), and GC content at the third position (GC3) for *CPP* families across eight species.

### 2.6 Evolutionary analysis of the *CPP* gene family

Collinearity analysis was performed using jcvi (v.1.4.23) (Tang et al., 2024), and DupGene_finder (Qiao et al., 2019) was used for duplicate gene classification. To identify syntenic blocks containing the *CPP* gene family, we filtered the collinearity results and then subsequently visualized using CIRCO*S* (v.0.69, https://circos.ca/). Ka/Ks calculations were performed using KaKs_Calculator (v.3.0) (Zhang, 2022) with the YN method.

### 2.7 Promoter analysis of the *CPP* gene family

We retrieved 2,000 base pairs (bp) of DNA sequence upstream of the ATG start codon from reference genomes and submitted them to the PlantCARE database (http://bioinformatice.psb.ugent.be/webtools/plantcare/) for putative cis-regulatory element identification.

### 2.8 Gene expression analysis

Expression profile analysis was conducted for 33 *CPP* genes from *C. chekiangoleosa*, *C. oleifera*, and *C. sinensis*, data obtained from the TPIA database (Gao et al., 2024). Heat maps were generated using R (version 4.4.1).

### 2.9 qRT-PCR analysis

RNA extraction from *C. oleifera* leaves was performed using the MiniBEST RNA Extraction Kit (Code No. 9769, TaKaRa). cDNA synthesis was carried out using the iScript™ cDNA Kit (Code No. RR036A, TaKaRa). *C. oleifera GAPDH* was selected as the internal reference gene (Gong et al., 2020). The qRT-PCR conditions followed Zheng et al. (2025), with pre-denaturation at 95°C for 30 s; followed by 40 cycles of denaturation at 95°C for 10 s; annealing at 55°C for 20 s; extension at 72°C for 30 s. Each reaction was performed in triplicate. Gene expression was calculated using the 2^−ΔΔCt^ method (Livak and Schmittgen, 2001). The primers used for qRT-PCR experiments are listed (**Supplementary Table S2**) Data were analyzed using Duncan’s multiple range test in SPSS 22.0 (p < 0.05), with significance denoted by letters a, b, and c.

## 3 Results

### 3.1 Identification *CPP* genes in six Theaceae species

A total of 65 *CPP* gene family members were identified across the six studied Theaceae species (**Supplementary Table S1**). *C. chekiangoleosa*, *C. crapnelliana*, *C. japonica*, *C. oleifera*, *C.sinens* and *Stewartia sinensis* possess 11, 10, 11, 12, 10, and 11 *CPP* genes, respectively (**Supplementary Figure S1**). The protein lengths varied among *CPP* genes, ranging from 409 (*CcheCPP11*) to 981 (*CcheCPP10*). Among them, *C. japonica* has the lowest median protein length of 584, which is higher than that of the non-*Camellia* species, rice, at 518. In contrast, *C. chekiangoleosa* boasts the highest median length 776. The molecular weight of the CPP protein ranges from 45.45 kDa (*CcheCPP11*) to 106.21 kDa (*CcheCPP10*), highlighting the diversity in protein size. The theoretical isoelectric point (pI) ranges from 4.91 (*CcheCPP7*) to 8.71 (*CcraCPP10*), with most proteins acidic and a few alkaline. These findings suggest that the Theaceae *CPP* gene family showed significant diversity in protein length, molecular weight, and isoelectric point. Subcellular localization predictions for all 65 CPP proteins consistently indicated their nuclear localization, suggesting that their primary functional role takes place within the nucleus.

### 3.2 Phylogenetic classification of *CPP* gene family

**Figure 1:**
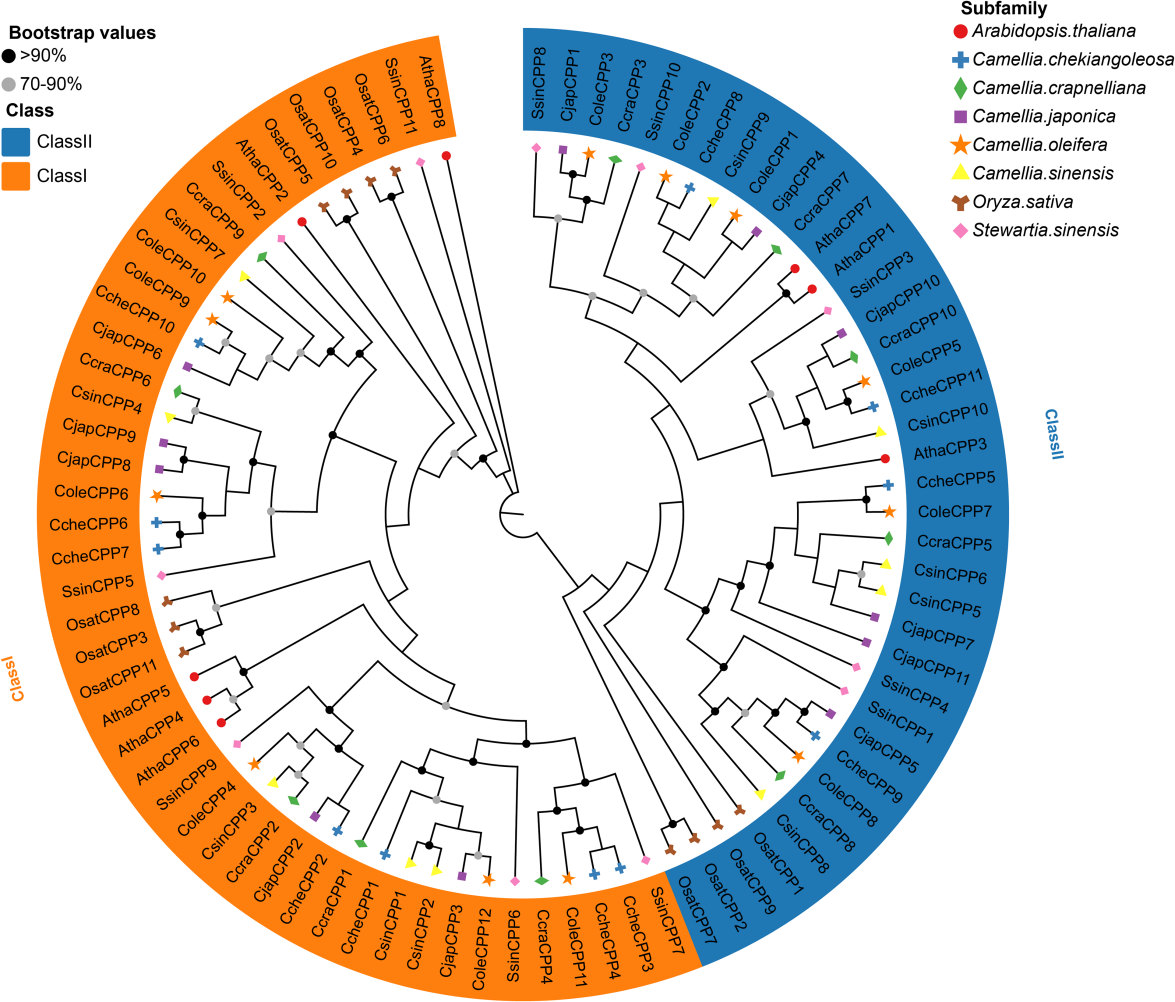
Phylogenetic ML tree of the *CPP* gene family from six Theaceae species, *Oryza sativa* and *Arabidopsis thaliana*. The different colored branches represent different subfamilies. Genes designated with Atha prefix correspond to *A. thaliana*, Cche is *C. chekiangoleosa*, Ccra is *C. crapnelliana*, Cjap is *C. japonica*, Cole is *C. oleifera*, Csin is *C. sinensis*, Osat is *O. sativa*, Ssin is *S. sinensis*.

To explore the evolutionary patterns of the *CPP* gene family in Theaceae species, this study constructed a maximum likelihood phylogenetic tree based on the CPP protein sequences of *Arabidopsis thaliana*, rice, and six Theaceae species (**Figure 1**, **Supplementary Table S1**). The topological structure clearly divides the *CPP* genes into two major groups: Class I and Class II, where Class I contains 34 members and Class II contains 31 members. While most of Theaceae species show a minimal difference (within one member) between the two classes, a significant imbalance was found in *C. chekiangoleosa* (7 Class I vs. 4 Class II). This skewed distribution parallels that in *A. thaliana* (5 vs. 3) and rice (7 vs. 4).

### 3.3 Multiple sequence alignment of *CPP* gene family

**Figure 2:**
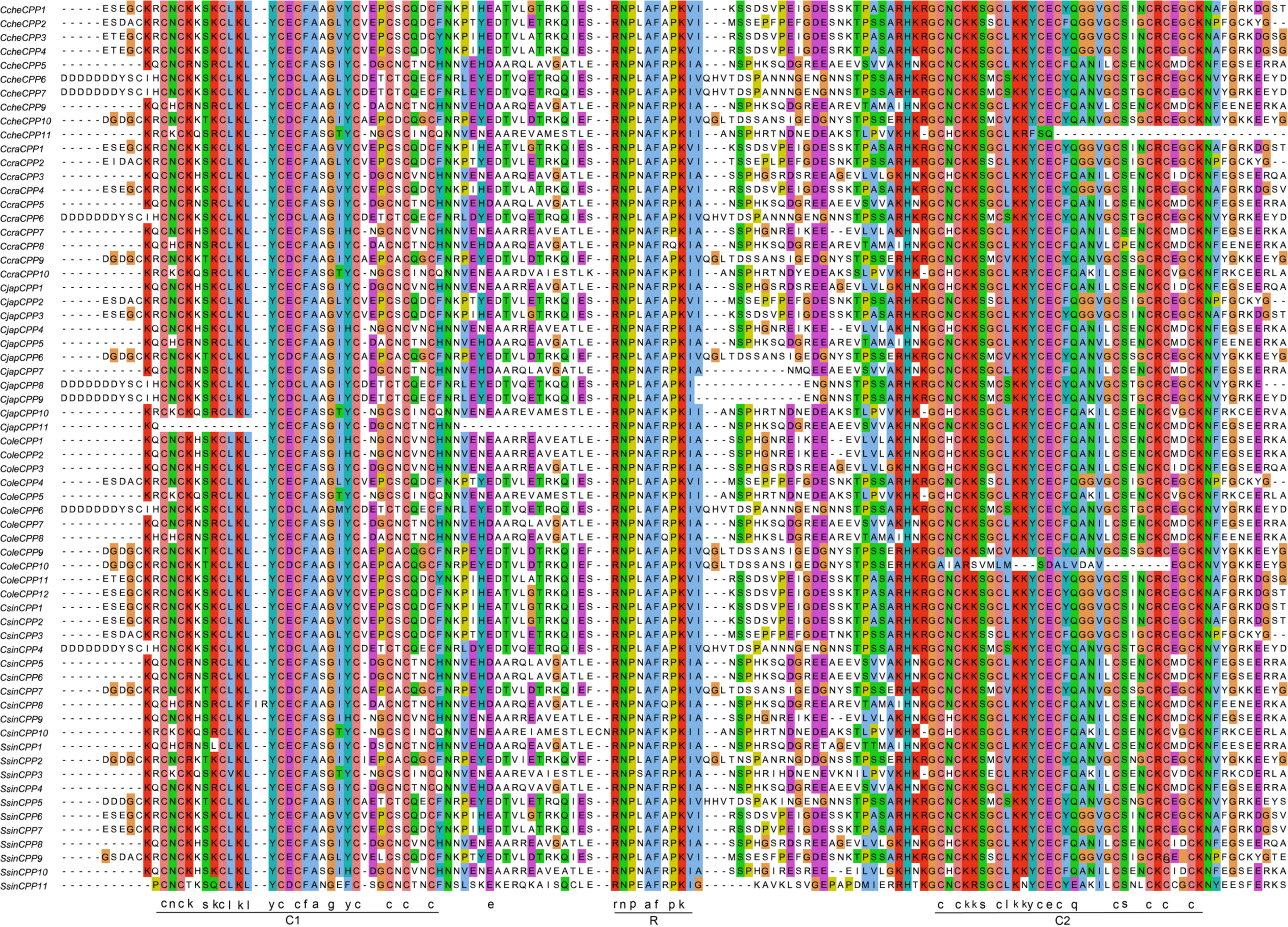
Multiple sequence alignment of the *CPP* gene family in Theaceae. C1 and C2 represent the conserved CXC domains, while R represents one conserved motif.

To further elucidate the conservation of protein sequences within the *CPP* gene family, a multiple sequence alignment was performed on the CPP protein sequences from six Theaceae species (**Figure 2**). The alignment revealed that all CPP family proteins are highly conserved and possess the characteristic C1-R-C2 domains. Nearly all members contain two cysteine-rich domains, C1 (CNCKXSXCLKLYCECFAXGXYCXEXCXCXNCXN) and C2 (CXCKKSXCLKKYCECFQXXVXCSXXCXCXXCKN), each with nine cysteines, demonstrating the family’s structural and functional stability (**Supplementary Figure S2**). The C1 domain showed slightly less conservation compared to the C2 domain. In some species, certain members of the CPP family have experienced loss or mutation in critical amino acids. For instance, *CjapCPP11* has mutations early in the C1 domain, resulting in the removal of its first 11 amino acids, including three cysteines. In contrast, *CCheCPP11* is missing the latter portion of the C2 domain and *ColeCPP10* has nearly lost the entire C2 domain.

Furthermore, one highly conserved R motif (RNPXAFXPK) located in the interval between C1 and C2, further underscores the conservation within the CPP family proteins. This motif maintained in the vast majority of CPP proteins, with only *CcraCPP8* had one mutation with proline (P) replaced by a glutamine (Q). This suggested that the motif was equally important in maintaining the structural integrity of CPP proteins and their functional regulation.

### 3.4 Gene structure and motif analysis of the *CPP* gene family

**Figure 3:**
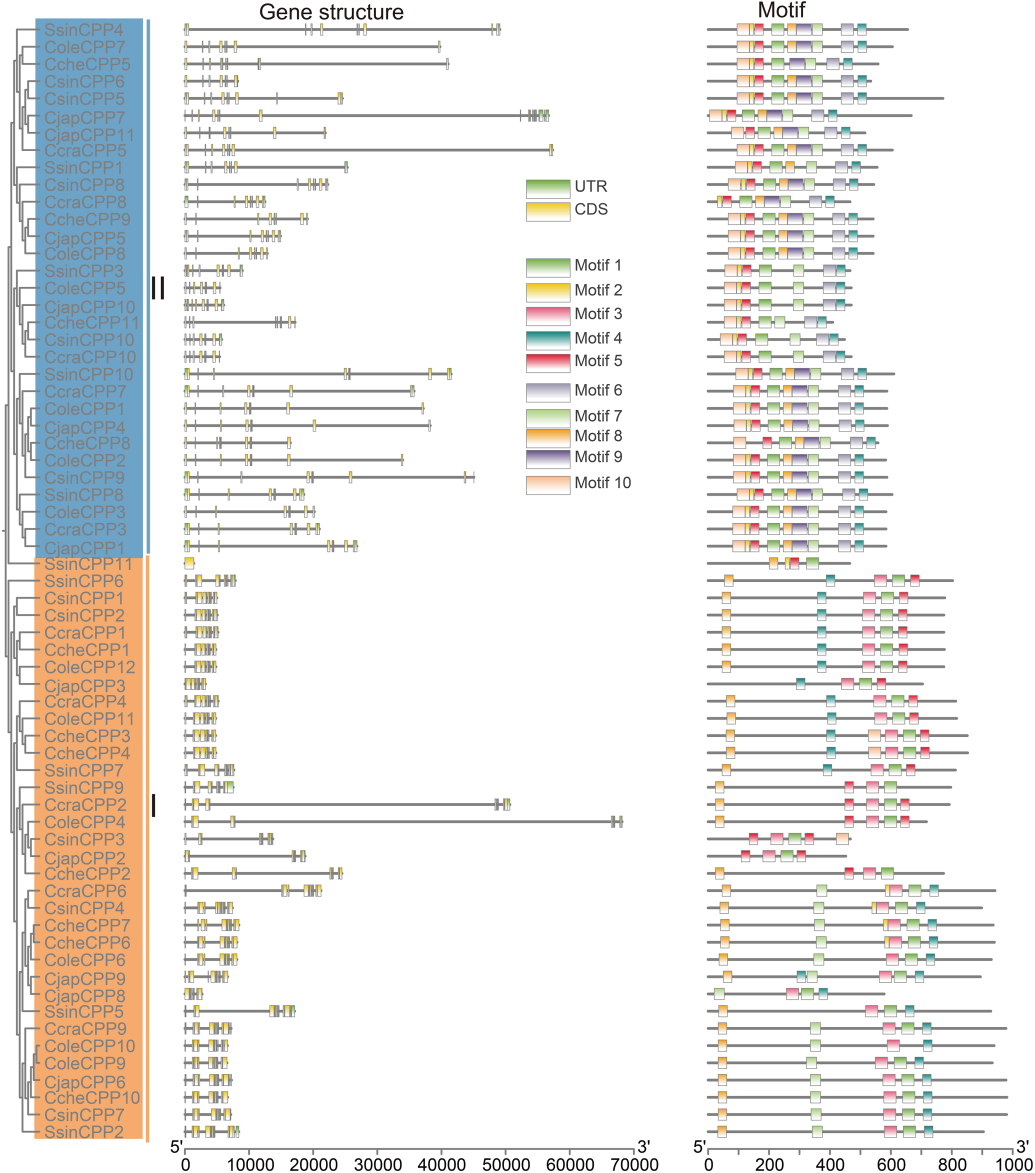
Gene structure and motifs analysis of the *CPP* gene family in Theaceae. On the left side is phylogenetic tree of *CPP* genes. Middle side is gene structure and motifs were in the right.

Gene structure differentiation plays a crucial role in the adaptive evolution of gene families. The two groups of *CPP* genes in six Theaceae species showed distinct exons numbers (**Figure 3**). In Class I *CPP* genes, the number of exons ranged from 1 (*SsinCPP11*) to 13 (*CjapCPP6*). 15 *CPP* genes (about 48.4%) have 10 exons, indicating that 10 exons were the main structural type of Class I genes. The rest of the *CPP* genes were distributed in intervals of 7-9 (about 29%) and 11-13 (about 22%) exons. There were also extreme cases, such as *SsinCPP11*, which contained only one exon, suggesting that it may have undergone structural simplification. Overall, Class I genes had a certain degree of diversity, but in general, they had a core pattern of 10 exons. In contrast, the number of exons in Class II *CPP* genes was more conserved, with the vast majority of genes having eight exons (22, or about 71%). Only a few genes showed a slight variation, with 9 or 10 exons. Notably, the *CjapCPP7* gene contained 17 exons, the largest number of exons in the class. Overall, the exon distribution of Class II *CPP* genes was highly concentrated, reflecting that they had maintained high structural stability and functional conservation during evolution. In addition, most *CPP* genes had UTR structures in the 5’ and 3’ non-coding regions.

In terms of protein motif analysis, a total of 10 conserved motifs were identified (**Figure 3**, **Supplementary Figure S3**). The phylogenetic tree results showed significant differences in the number and distribution pattern of motifs among different groups of genes, and genes within the same subfamily usually exhibit relatively consistent motifs composition and arrangement characteristics. The motif architecture differed significantly between the two CPP classes. While class I genes were characterized by a limited repertoire of two predominant motif arrays (motif 8-4-3-1–5 and 8-7-3-1-4), class II genes presented a striking contrast in their conservation. The vast majority of Class II members shared an invariant repertoire of nine motifs (motif 10, 2, 5, 1, 8, 9, 7, 6, and 4), suggesting stronger evolutionary constraints. Specifically, in class II, the motifs 10, 2, 5, and 1 were associated with two CXC domains, whereas in class I, the motifs Motif 3 and 1 were involved.

### 3.5 Codon preference analysis of the *CPP* gene family

The comparative analysis of six Theaceae plants *CPP* genes further revealed significant differences in codon usage bias and genetic characteristics, highlighting the intrinsic diversity of the family and its specific evolutionary adaptability. RSCU (Relative Synonymous Codon Usage), a key metric for quantifying CUB (Codon Usage Bias), measures by comparing the observed frequency of each synonymous codon with the expected frequency under equal usage. Analysis of RSCU values for the *CPP* gene family (**Supplementary Figure S4A**) identified two distinct groups of codon usage frequencies, termed group I and group II. Group I comprised 22 codons with high usage frequencies (average RSCU exceeding 1.16), while group II included 42 codons with lower usage frequencies (all below 1). Notably, the codons UUG, GCU, GUU, UCU, AGG, and AGA had the highest usage frequencies, with RSCU values all surpassing 1.5.

The codon usage bias of *CPP* gene family was further investigated by analyzing the Codon Adaptation Index (CAI), Effective Number of Codons (ENC), GC, GC1, GC2, and GC3 content (**Supplementary Figure S4B**). The CAI exhibited minimal variation across different Theaceae species, with average values clustering around 0.68. This was significantly lower than that of the monocot rice. The ENC showed some variation within Theaceae family, with the lowest value observed in *C. chekiangoleosa* (50.94) and the highest in *S. sinensis* (52.37). Despite this variation, the overall preference within the Theaceae appeared relatively weak and lower than that of rice. The GC content also displayed minimal fluctuation across Theaceae species, with average values centered around 0.43-0.44, similar to *A. thaliana* but significantly lower than the total GC content of monocot rice (0.48). This underscores the stability of GC content in Theaceae and dicot plants in general. Further analysis revealed that while GC1 and GC2 sites showing similar patterns across species, the divergence between Theaceae and rice was most pronounced at the GC3 site. The average GC3 values for Theaceae and *A. thaliana* species concentrated around 0.37, whereas rice had a higher average of 0.46, indicating a clear divergence between monocots and dicots at this site. These observations likely reflect the conserved genomic features among Theaceae species, which were distinct from those of monocot plants.

### 3.6 Chromosome distribution and duplication analysis in Theaceae

**Figure 4:**
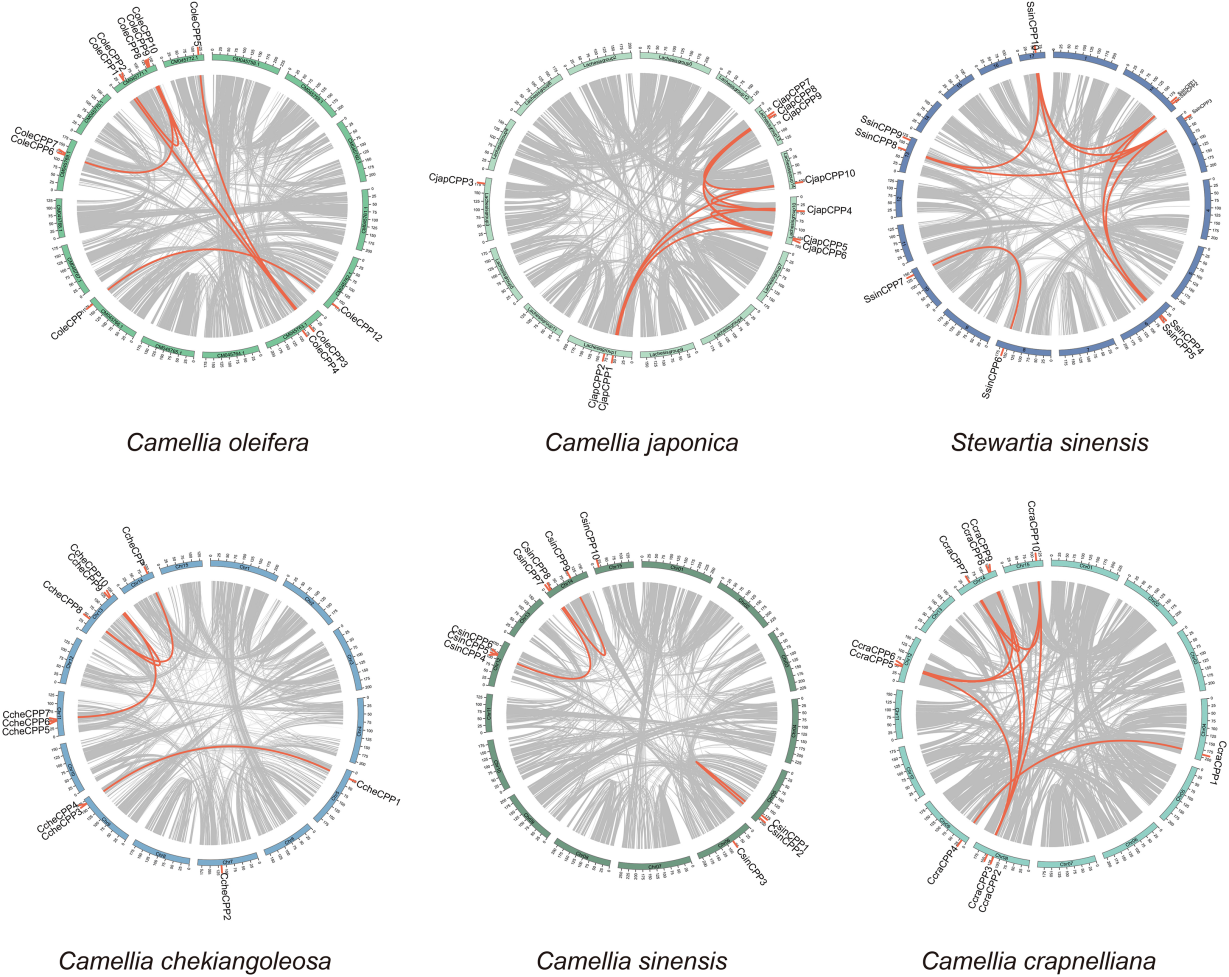
Collinearity analyses of *CPP* gene family in six Theaceae plants. Red links highlight segmental duplication of *CPP* gene pairs.

The chromosome distribution map reveals that when the distance between two *CPP* genes was limited to 1–2 megabases (Mb), multiple clusters of *CPP* genes had been identified in three species (**Figure 4**). In *C. chekiangoleosa*, two gene clusters were formed (*CcheCPP3* and *CcheCPP4* on chromosome 9, and *CcheCPP6* and *CcheCPP7* on chromosome 11). *C. oleifera* harbors two clusters, located on chromosome 13, comprising *ColeCPP1* and *ColeCPP2*, as well as *ColeCPP9* and *ColeCPP10*. *C. japonica* features only one single cluster positioned on chromosome 13, consisting of *CjapCPP8* and *CjapCPP9*.

Intraspecific collinearity results (**Figure 4**, **Supplementary Table S3**) indicated that in *C. lanceoleosa* and *C. oleifera*, seven pairs of collinear *CPP* genes were detected respectively. In *C. japonica* and *C. crapnelliana*, nine pairs were found each. Four pairs in *C. chekiangoleosa*, three pairs in *C. sinensis*, and seven pairs in *C. oleifera* were observed, respectively, while in *S. sinensis*, ten pairs were detected. By conducting a comparative analysis of the nonsynonymous substitution rate (Ka), synonymous substitution rate (Ks), and the Ka/Ks ratio among different species (**Supplementary Figure S5**), we found that the Ka and Ks values of rice and *A. thaliana* were higher than those of Theaceae species. However, the Ka/Ks ratio of all species were below 1, with an average value of 0.32. This result indicated that the *CPP* genes were generally subject to strong purifying selection. There were also certain differences among different species. In the Theaceae family, the Ka/Ks ratio of all species was higher than that of *A. thaliana*, with *C. japonica* being the most significant. This suggests that there were differences in the selective pressures experienced by different species, and that Theaceae species may be subject to relatively relaxed selective pressure.

**Figure 5:**
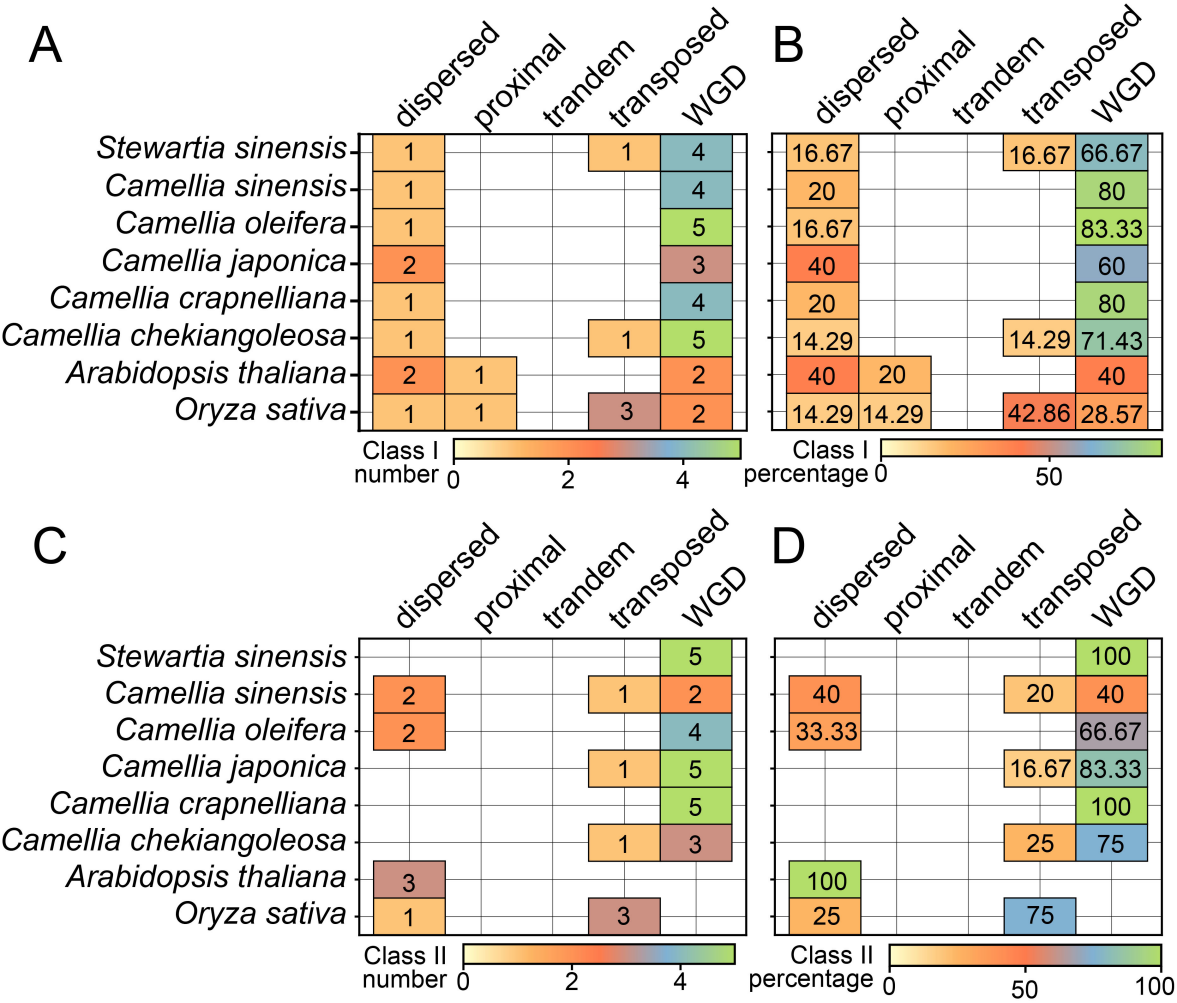
The distribution of *CPP* genes derived from various duplication modes. The number **(A)** and percentage **(B)** distributions from class I *CPP* genes. The number **(C)** and percentage **(D)** distributions from class II *CPP* genes.

Our analysis of the five gene duplication types—whole genome duplication (WGD), proximal, tandem, dispersed, and transposed—revealed that WGD was the most prevalent in both Class I and Class II, accounting for 60–88.33% and 40–100% of genes, respectively (**Figure 5**). This finding aligns with the collinearity results depicted in **Figure 4**. Specifically, in Class I, the WGD proportion in all Theaceae species exceeded that of rice and *A. thaliana*, especially in *C. crapnelliana* and *C. oleifera*, where *C. sinensis* had over 80% of genes (**Figures 5A, B**). In Class II, rice and *A. thaliana* had no WGD type, while all Theaceae species had WGD-derived genes, especially in *C. crapnelliana* and *S. sinensis*, all of which originated from WGD genes (**Figures 5C, D**). This indicated that WGD was the main driving force the expansion of the *CPP* gene family, particularly in promoting the expansion of genes in Class II *CPP* genes.

### 3.7 Collinearity analysis of *CPP* genes across species of Theaceae family

**Figure 6:**
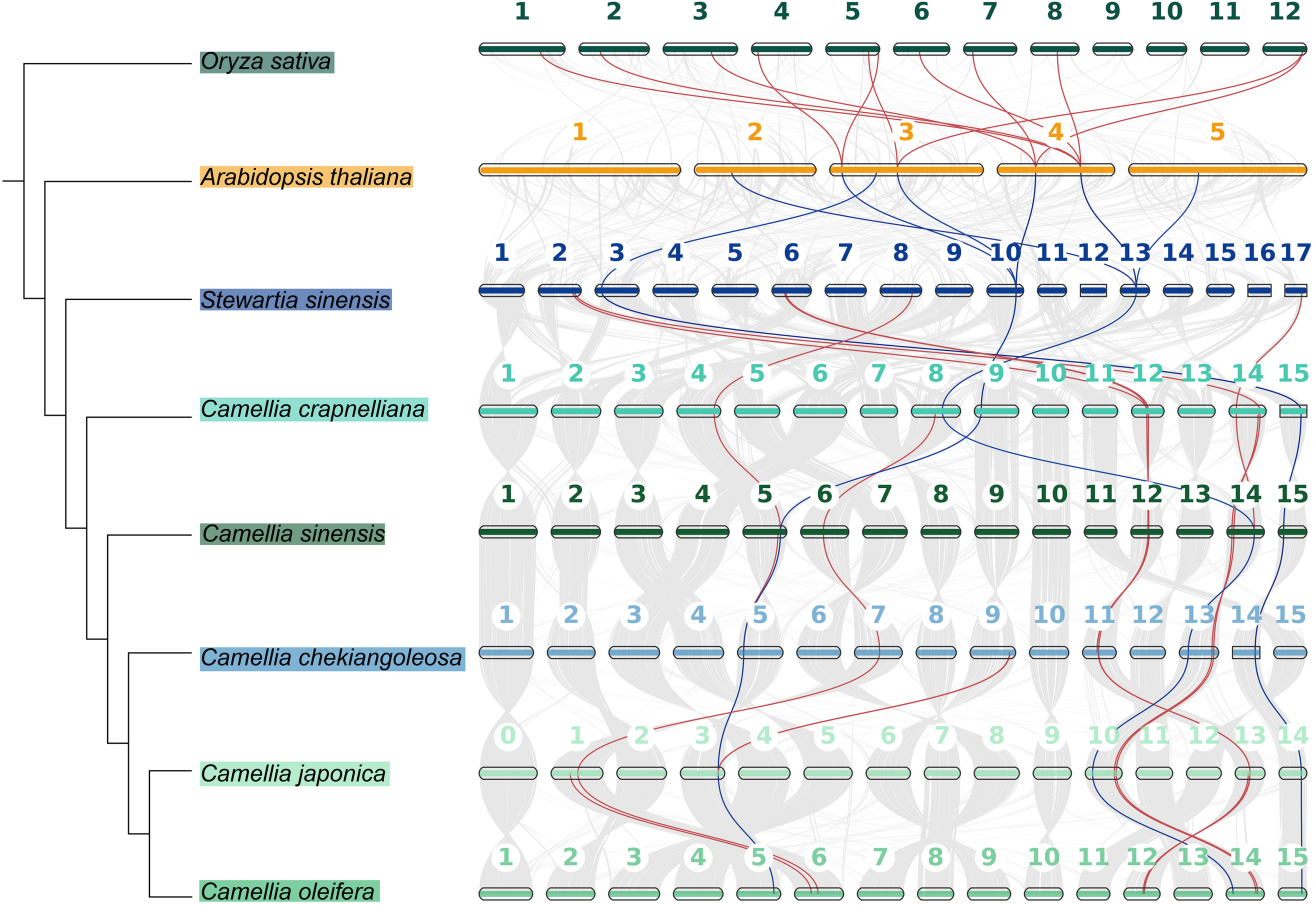
The collinearity analysis of *CPP* family genes across six Theaceae and *A.thaliana* and *Oryza sativa*. Red lines represent collinear *CPP* gene pairs between Theaceae and two other species and the distinctive blue lines indicate genes that exhibit collinearity with *A. thaliana*.

In order to explore the evolutionary relationships of the *CPP* genes in different species of the Theaceae family, we conducted a collinearity analysis (**Figure 6**, **Supplementary Table S4**). The results showed that the *CPP* genes among Theaceae species had a conserved synteny relationship. Specifically, there were 11 collinear pairs between *S. sinensis* and *C. crapnelliana*, 10 pairs between *C. crapnelliana* and *C. sinensis*, 10 pairs between *C. sinensis* and *C. chekiangoleosa*, 11 pairs between *C. chekiangoleosa* and *C. japonica*, and 11 pairs between *C. japonica* and *C. oleifera*. These results indicated that the *CPP* genes among Theaceae species had maintained a high degree of conservation during the evolutionary process. In addition, we also observed that there are 8 collinear pairs between the earliest Theaceae species *S. sinensis* and *A. thaliana*, including three genes *SsinCPP3*, *SsinCPP7*, and *SsinCPP8*, which were homologous to *ColeCPP5*, *ColeCPP12*, and *ColeCPP1*, respectively. This suggests that these three genes were relatively conserved among dicotyledonous plants. Among them, *SsinCPP7* and *SsinCPP8* also had collinearity with rice, further implying that these two genes are more conserved.

### 3.8 Promoter analysis of the *CPP* gene family

**Figure 7:**
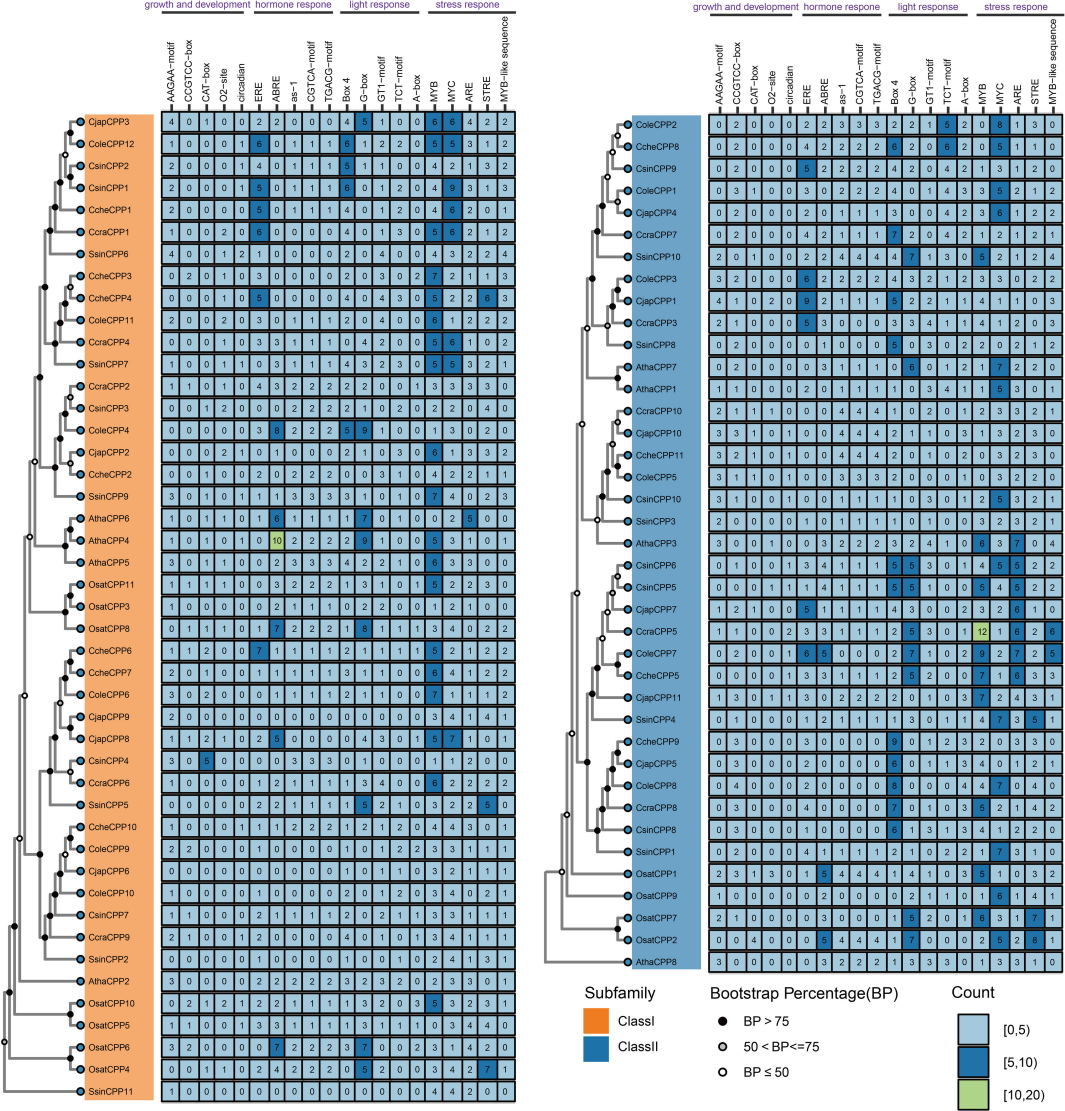
Distribution of the top five promoter elements in each category within *CPP* genes.

A total of 2,886 instances of 82 types of cis-acting elements were identified from 65 *CPP* genes of six Theaceae species (**Supplementary Table S5**). Among these, *CcraCPP5* harbored the highest number of cis-acting elements (72), followed by *ColeCPP7* and *SsinCPP10*, each containing 69 and 62 elements, respectively. The cis-acting elements were categorized into four functional groups based on their roles: plant growth and development (244 elements), hormone response (651 elements), light response (807 elements), and stress response (1184 elements) (**Supplementary Table S5**). Notably, stress response elements accounted for 41.03%, making it the predominant category. As shown in **Figure 7**, the top five most abundant elements in each category were highlighted, revealing that MYB, MYC, ARE, and STRE were the most common in plant response elements. This suggests that *CPP* genes may play a crucial role in the stress response of Theaceae plants. Additionally, the abundance of light response elements such as G-box and Box4 indicates that *CPP* genes also significantly contribute to the growth and development of Theaceae plants.

### 3.9 Expression patterns of CPP genes in different tissues and treatment

**Figure 8:**
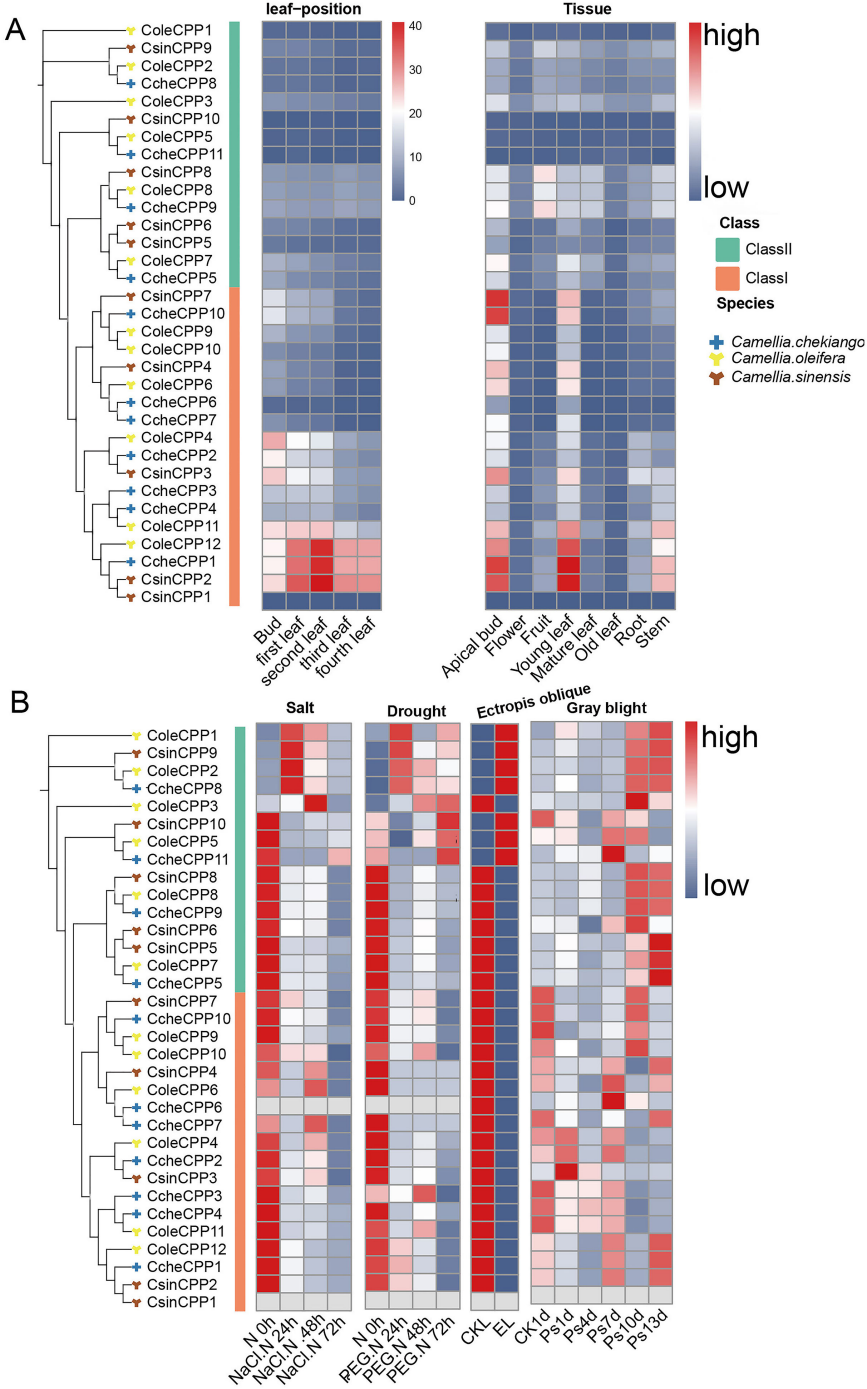
Expression of *CPP* genes in under different tissues and stresses treatment in three Theaceae plants. The left side features an evolutionary tree, while the right side displays an expression heatmap. Genes designated with Cole prefix correspond to *C*. *oleifera*, Cche is *C. chekiangoleos*, Csin is *C*. *sinensis.***(A)** for different tissues and **(B)** for different stress treatment.

The transcriptome landscape constructed using RNA-seq data has revealed the expression patterns of *CPP* genes in three Theaceae species (*C. oleifera*, *C. chekiangoleosa* and *C. sinensis*) under various tissues and stress conditions (**Figure 8A**). In different leaf position samples, Class I *CPP* genes exhibited significantly high expression levels, such as *CsinCPP2*, *CcheCPP1*, and *ColeCPP12*, with the highest expression in the second leaf. Further analysis of different tissue parts showed that these three genes were also highly expressed, mainly in the apical bud, young leaf, and stem. Additionally, *CsinCPP7* and *CcheCPP7* also showed high expression in the apical bud. It is noteworthy that although some genes cluster together on the evolutionary tree, their expression patterns were not the same. For example, *CsinCPP1* was clustered together with *CsinCPP2* on the evolutionary tree, have lower expression levels in leaf-position samples, indicating differences in gene expression among different Theaceae species. We further investigated the expression patterns of *CPP* genes under various stress conditions (**Figure 8B**). A group of genes in Class II, including *ColeCPP1*, *CsinCPP9*, *ColeCPP2*, *CcheCPP8*, were significantly upregulated under stress conditions such as drought, salt stress, *Ectropis oblique*, and gray blight. Overall, these results highlight the crucial role of *CPP* genes in mediating the responses of Theaceae plants to both biotic and abiotic stresses.

### 3.10 Validation of *CPP* gene expression in *Camellia oleifera* under drought and salt stress treatment

**Figure 9:**
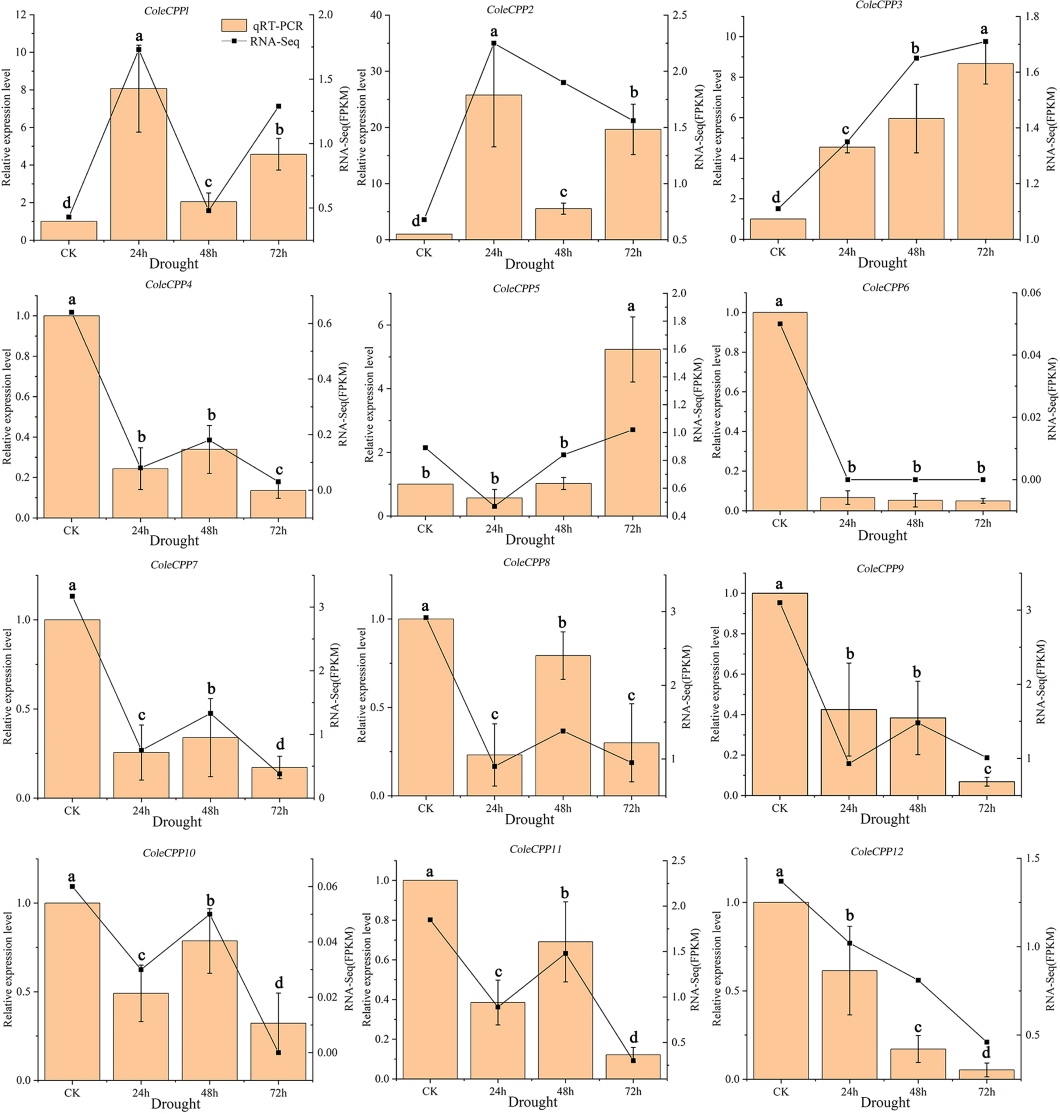
Relative expression patterns of the *CPP* gene family under drought stress in *camellia oleifera*. The bar chart represents the qRT-PCR quantification results, while the line chart represents the transcriptome quantification results. Data were the mean of 3 independent biological replicates. Different letters above the bar chart indicated significant differences at *P* < 0.05. (Error bars indicate the standard error (SE) between three replicates).

Under drought stress, the expression levels of 12 *CPP* genes in *C. oleifera* leaf tissues were determined by qRT-PCR, and the expression levels in the control leaves were standardized to 1 (**Figure 9**). *ColeCPP4*, *ColeCPP6*, *ColeCPP9*, *ColeCPP12* and *ColeCPP7* were consistently downregulated, reaching a minimum at 72 hours. In contrast, *ColeCPP8*, *ColeCPP10* and *ColeCPP11* showed a trend of first falling and then recovering (**Figure 9**). In Class II, *ColeCPP1* and *ColeCPP2* showed a similar upregulated expression pattern, peaking at 24 hours (8.07 times and 25.76 times the control, respectively) and then declining. *ColeCPP3* and *ColeCPP5* were gradually upregulated with the extension of drought duration, reaching a peak at 72 hours (8.67 times and 5.23 times, respectively). These findings were similar with the results obtained from transcriptome analysis.

**Figure 10:**
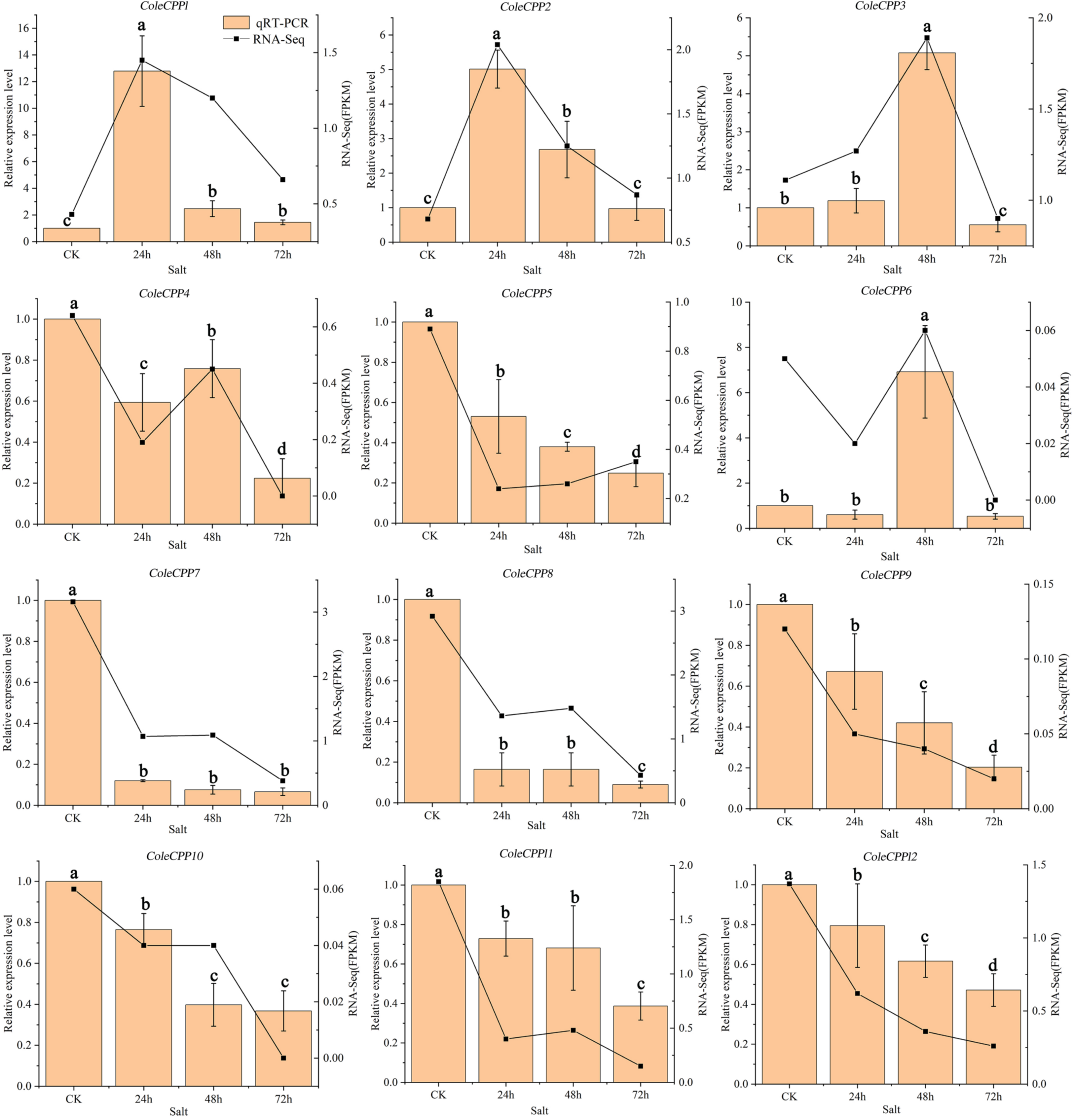
Relative expression patterns of the *CPP* gene family under salt stress in *camellia oleifera*. The bar chart represents the qRT-PCR quantification results, while the line chart represents the transcriptome quantification results. Data were the mean of 3 independent biological replicates. Different letters above the bar chart indicate significant differences at *P* < 0.05(Error bars indicate the standard error (SE) between three replicates).

The expressions of the 12 *CPP* genes were also validated by qRT-PCR under salt stress (**Figure 10**). Class II genes *ColeCPP5*, *ColeCPP7*, *ColeCPP8* and *ColeCPP11*, as well as Class I genes *ColeCPP9*, *ColeCPP10* and *ColeCPP12*, were consistently downregulated and reached their minimum at 72 hours. *ColeCPP4* showed a pattern - initial down-regulation, brief up-regulation and final down-regulation - and finally reached its lowest level at 72 hours. *ColeCPP1* and *ColeCPP2* were significant upregulated in salt stress and peaked at 24 hours (12.79 times and 5.01 times the control, respectively) and then declined. *ColeCPP3* and *ColeCPP6* were also upregulated peaked at 48 hours, at 5.08 times and 6.92 times respectively. These findings aligned closely with those derived from transcriptome analyses.

## 4 Discussion

The *CPP* gene family plays crucial roles in plant development. With the rapid advancement of bioinformatics, identification and analysis of *CPP* gene families have been completed in numerous species. Related studies identified 8 members in *A. thaliana* (Yang et al., 2008), 11 in *Oryza sativa* (Yang et al., 2008), 20 in soybean (Zhang et al., 2015), 13 in maize (Song et al., 2016), 5 in cucumber (Zhou et al., 2018), 11 in *C. sinensis* (Yang et al., 2019), 20 in *Gossypium hirsutum* (Huang et al., 2022), and 10 in *Mangifera indica* (Yang et al., 2021). Within the Theaceae family, the number of *CPP* family members were similar across different plants, typically 11, except for 10 in *C. crapnelliana* and 12 in *C. oleifera*. The number of *CPP* genes in Theaceae species was similar to *Oryza sativa* but greater than in *A. thaliana*, possibly due to WGD events. Further bioinformatic analysis revealed that *CPP* genes exhibit pronounced diversity in physicochemical properties—including length, molecular weight, and isoelectric point, a characteristic largely consistent with the *CPP* gene family in Moso bamboo (Tan et al., 2024).

Phylogenetic analysis divided the *CPP* gene family into two subclasses (I-II). According to the phylogenetic tree analysis, genes clustering closer together likely shared similar functions and structures. For example, studies on *Phoebe bournei* had indicated that CPP proteins can be divided into several conserved subgroups, which were unevenly distributed across different species (Liu et al., 2025). Similarly, phylogenetic analysis in soybean had revealed that certain subgroup members had expanded in specific species, suggesting that the evolution of the *CPP* gene family may be associated with species-specific adaptations. Furthermore, research in maize had also identified distinct subgroup-specific expansions of *CPP* genes, which were likely closely related to gene duplication events. In combination with the findings of this study, it could be speculated that the expansion of *CPP* genes in Theaceae, which may be associated with whole-genome duplication (WGD) events or segmental duplication, thereby enhancing the functional diversity of this gene class in Theaceae plants under evolution. In addition, we also revealed that although multiple *CPP* genes are clustered on chromosomes (**Figure 4**), they are not closely adjacent but rather distributed across a certain interval 1-2Mb region. This distribution pattern suggests that these gene clusters may be evolutionary relics of ancient tandem duplications, with additional genes inserted during chromosomal rearrangement (Panchy et al., 2016). Exon-intron architecture analysis showed that most of Class I and Class II genes contain multiple exons, except for *SsinCPP11*. Genes within the same clade exhibit similar exon numbers and gene lengths, whereas pronounced difference exist between subfamilies, implying structural constraints during their evolutionary history. Conserved sequence analysis revealed that CPP family proteins are highly conserved in Theaceae and feature C1 and C2 domains (**Figure 2**), each containing nine cysteine residues that form disulfide bonds essential for their structural integrity and function, thereby corroborating previous findings on this protein family (Lu et al., 2013). Collinearity analysis revealed numerous syntenic genes within Theaceae species. Further combining phylogenetic relationships and collinearity analysis showed that Theaceae plants shared closer evolutionary relationships with the dicot *A. thaliana* than with the monocot *Oryza sativa*. This finding aligned with the known evolutionary relationships among *C. sinensis*, *Oryza sativa* and *A. thaliana* (Wu et al., 2024), indicating that *CPP* genes underwent differentiation during the evolution of monocots and dicots in angiosperms. Codon usage bias analysis revealed significant two distinct codon usage patterns. The pronounced differences in codon preference between Theaceae *CPP* genes and those of model plants such as *A. thaliana* and *O. sativa* likely reflected divergent genomic backgrounds and expression regulation strategies that emerged through adaptation to distinct ecological niches over evolutionary time.

Cis-regulatory elements play essential roles in plant growth, development, hormone response, and stress response. *CPP* genes in Theaceae contained various regulatory elements primarily related to plant growth and development, hormone response, light response, and stress response. Among plant response regulatory elements, stress response-related elements were most abundant. All *CPP* genes contained stress response elements, indicating that *CPP* members likely played important roles in Theaceae plants’ response to biotic or abiotic stress. Additionally, the abundance of light response-related regulatory elements suggested a close relationship between *Camellia CPP* gene family and plant light response, consistent with *G. hirsutum CPP* gene family (Huang et al., 2022). Regarding hormone regulation, plant hormones such as abscisic acid (Liu et al., 2022), gibberellin, and auxin played crucial roles in salt-alkali stress response, thereby enhancing *Camellia*’s resistance to abiotic stress (Yang et al., 2023).

The expression profiles of genes can reflect their underlying biological functions and regulatory mechanisms. Heat map analysis revealed distinct expression patterns of *CPP* genes across different *Camellia* tissues. Most Class II *CPP* genes exhibited low expression levels throughout the developmental process from sprouting to fourth leaf emergence, as well as in roots and stems. In contrast, most Class I *CPP* genes demonstrated high expression levels during this developmental process and across various tissue types, including roots and stems. This difference may relate to functional variations and temporal specificity of *CPP* activities. *CPP* genes showed expression in multiple tissues, including apical buds, young leaves, roots, and stems, with notably higher expression in apical buds, young leaves, and stems compared to flowers and mature leaves, suggesting important regulatory roles in these tissues. While wheat *CPP* genes showed higher expression in roots and stems compared to leaves (Liu et al., 2023), Theaceae *CPP* genes exhibited higher expression in young leaves than in roots and stems, indicating tissue-specific expression patterns and potential neofunctionalization of certain genes.

The transcriptome analysis of the *CPP* gene family in three Theaceae species revealed distinct expression patterns across different tissues and under various stress conditions. Class I *CPP* genes, such as *CsinCPP2*, *CcheCPP1*, and *ColeCPP12*, exhibited high expression in leaves, apical buds, and stems, suggesting roles in growth and development. In contrast, several Class II CPP genes were significantly upregulated under stress conditions, indicating their involvement in stress responses. qRT-PCR validation in *C. oleifera* under drought and salt stress further supported these findings. These results highlighted the multifaceted roles of *CPP* genes in Theaceae plants, with Class I genes primarily involved in developmental processes and Class II genes playing crucial roles in stress tolerance. The distinct expression patterns underscore the functional diversity of the *CPP* gene family and their importance in maintaining plant growth and development under adverse conditions.

## 5 Conclusion

This study conducted a systematic investigation of the *CPP* gene family across six Theaceae species, identifying 65 *CPP* family members. Phylogenetic analysis classified *CPP*s into two subfamilies. Cis-regulatory element analysis revealed *CPP* genes’ primary involvement in stress response. Analysis of collinear gene pair duplication types across six genomes suggested WGD as the main evolutionary driver, with no observed tandem duplication. Transcriptome sequencing data and qRT-PCR analysis demonstrated the significant regulatory role of *Camellia CPP* genes in drought and salt stress responses. This comprehensive analysis provides evidence for structural and functional conservation of *CPP* genes in Theaceae plants and offered new perspectives on the adaptive evolution of the *CPP* gene family in perennial plant evolutionary history.

## Data Availability

The original contributions presented in the study are included in the article/**Supplementary Material**. Further inquiries can be directed to the corresponding authors.

## Supplementary material

Supplementary Figure 1 Physicochemical properties and distribution patterns of *CPP* genes.

Supplementary Figure 2 Multiple sequence alignment of the CPP gene family in Theaceae, *A. thaliana* and *O. sativa*.

Supplementary Figure 3 Sequence logo for each motif in *CPP* genes.

Supplementary Figure 4 Codon preference analysis of the *CPP* genes. **(A)** Analysis of relative synonymous codon usage (RSCU) in the *CPP* gene family. Each row represents a *CPP* gene from the indicated species, and each column represents codons. **(B)** The codon usage bias six parameters.

Supplementary Figure 5 The Ka, Ks, and Ka/Ks of *CPP* genes among Theaceae, *A. thaliana* and *O. sativa*.

Supplementary Table 1 The *CPP* genes identified in this study.

Supplementary Table 2 Primer sequences used for qRT-PCR validation of candidate genes.

Supplementary Table 3 Intra-collinear gene pairs identified in six Theaceae species.

Supplementary Table 4 Collinear gene pairs among different species.

Supplementary Table 5 Cis elements identified in *CPP* genes.
