# Supplementary figures and images for: Genome-wide identification, evolution and expression of the CPP gene family in six Theaceae species
Source: Front Plant Sci. 2025 Nov 10;16:1700390. doi: 10.3389/fpls.2025.1700390 (PMC12643006; doi:10.3389/fpls.2025.1700390)

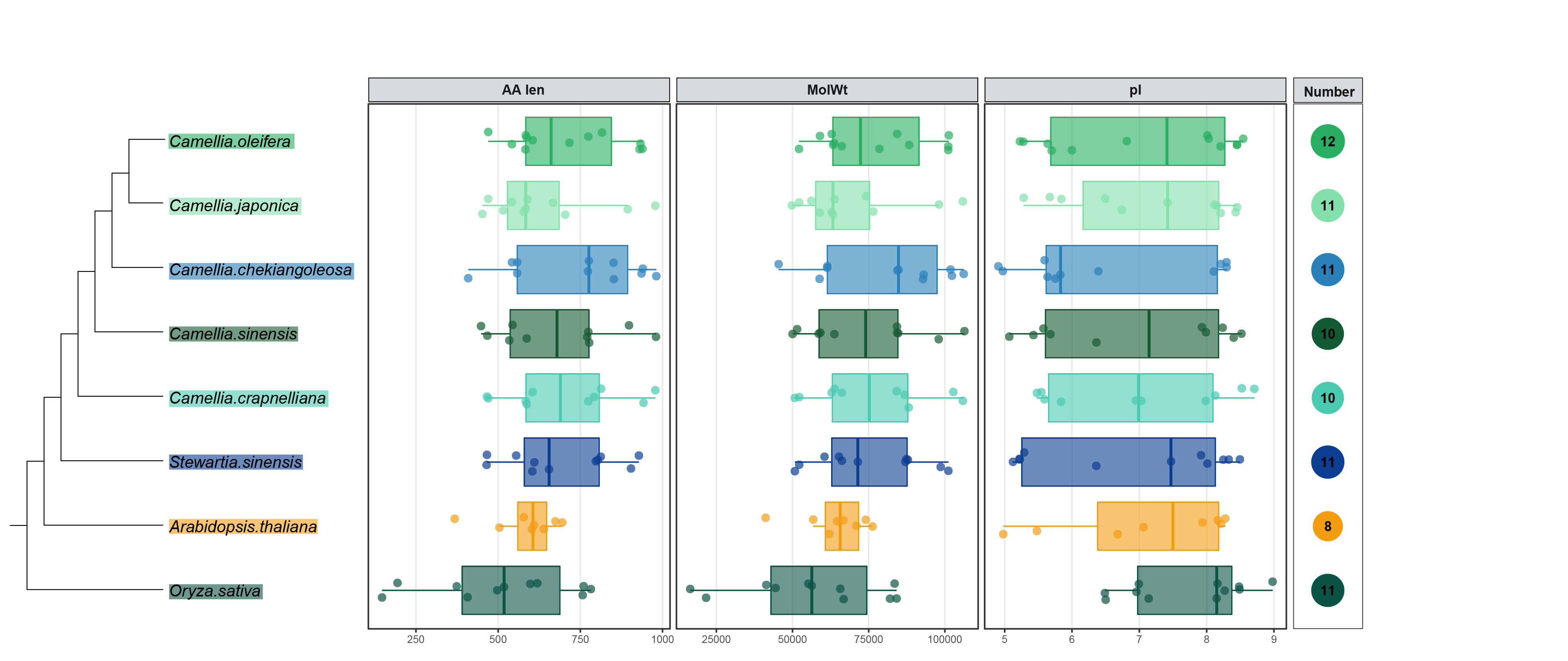

Supplement: Supplementary Figure 1 — Physicochemical properties and distribution patterns of CPP genes. [file Image1.jpeg]

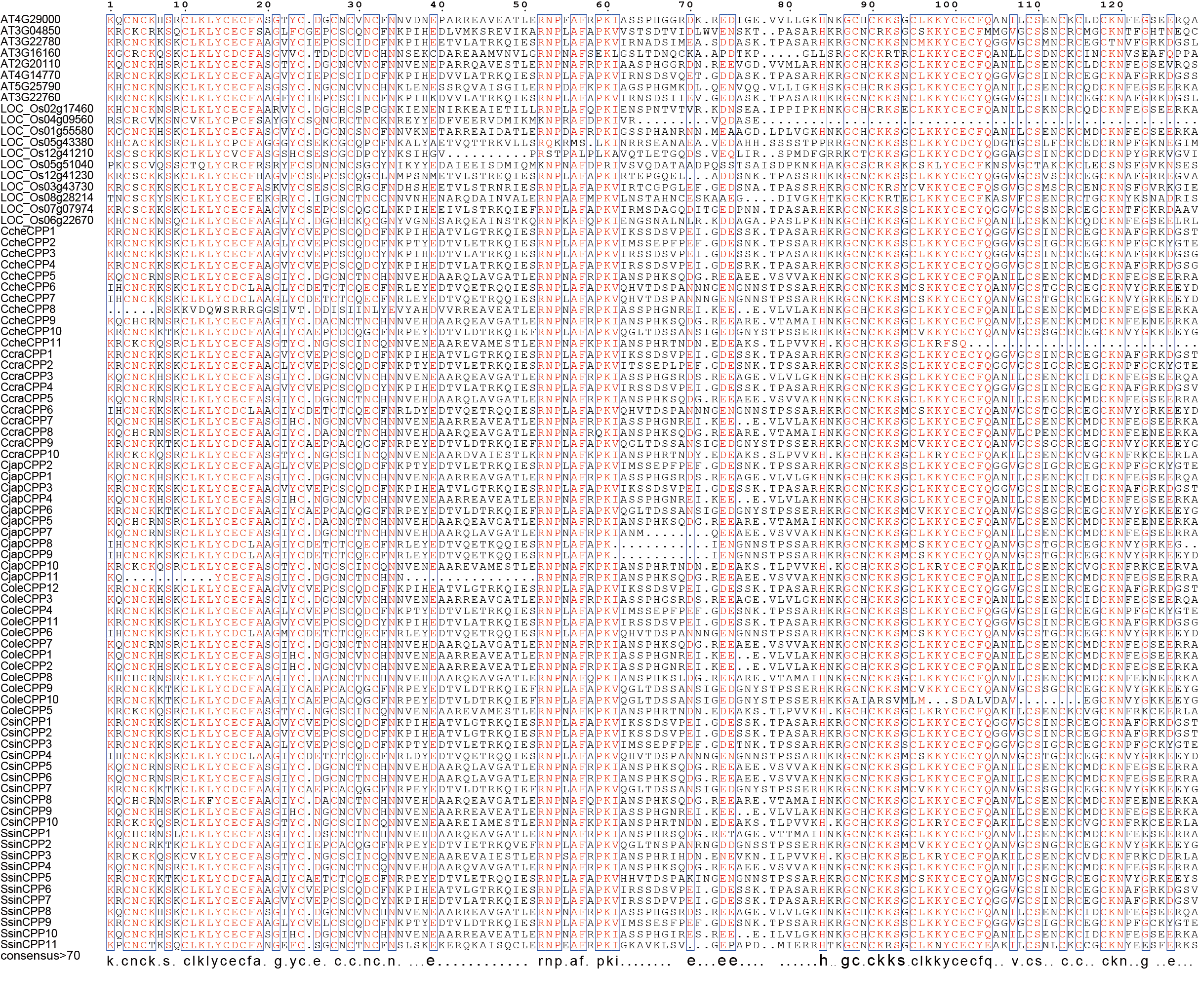

Supplement: Supplementary Figure 2 — Multiple sequence alignment of the CPP gene family in Theaceae, A. thaliana and O. sativa. [file Image2.png]

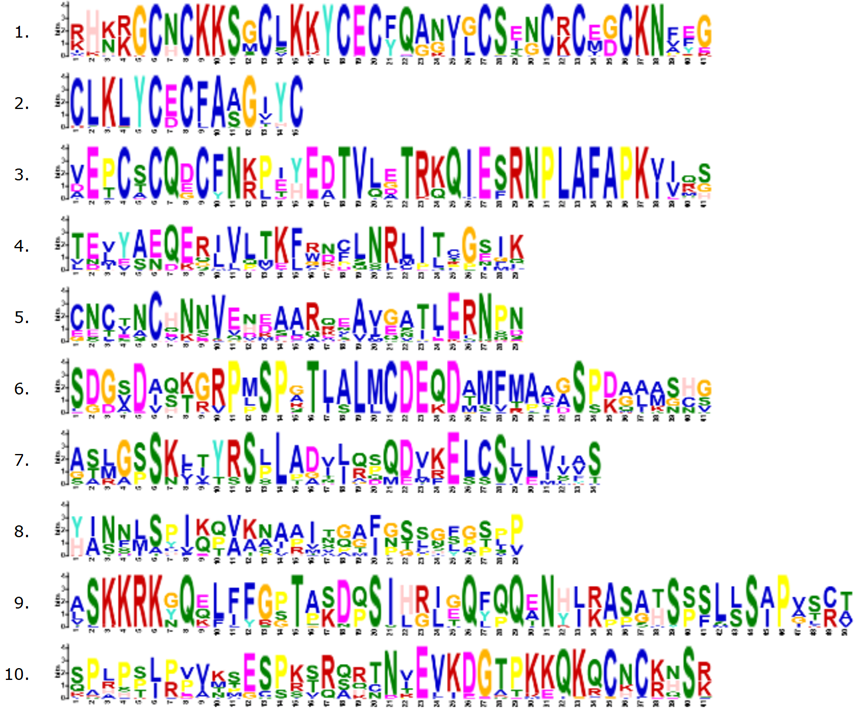

Supplement: Supplementary Figure 3 — Sequence logo for each motif in CPP genes. [file Image3.jpeg]

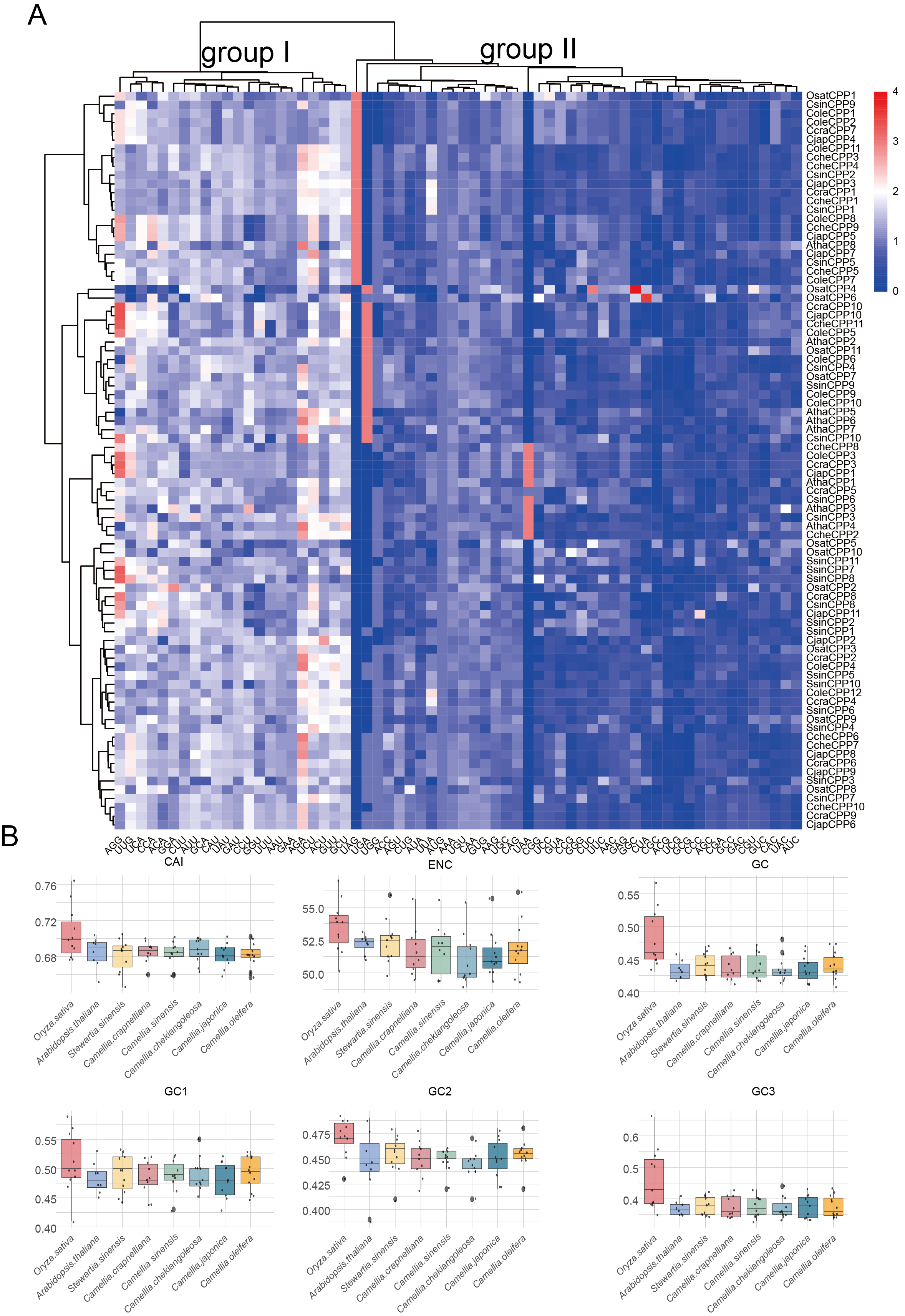

Supplement: Supplementary Figure 4 — Codon preference analysis of the CPP genes. (A) Analysis of relative synonymous codon usage (RSCU) in the CPP gene family. Each row represents a CPP gene from the indicated species, and each column represents codons. (B) The codon usage bias six parameters. [file Image4.jpeg]

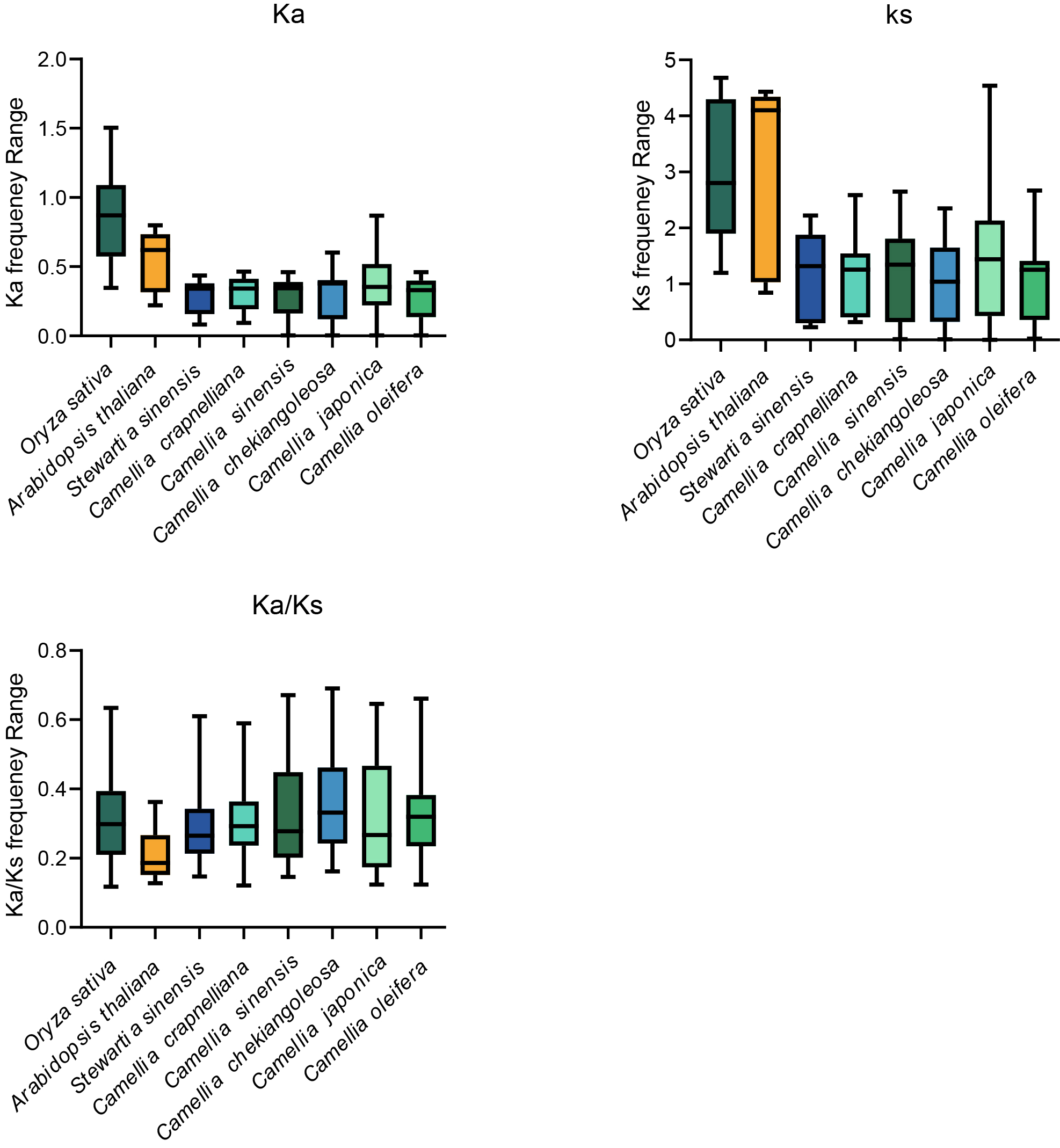

Supplement: Supplementary Figure 5 — The Ka, Ks, and Ka/Ks of CPP genes among Theaceae, A. thaliana and O. sativa. [file Image5.jpeg]
